# Novel microRNA families expanded in the human genome

**DOI:** 10.1186/1471-2164-14-98

**Published:** 2013-02-12

**Authors:** Zhi-Qiang Du, Cai-Xia Yang, Max F Rothschild, Jason W Ross

**Affiliations:** 1 Department of Animal Science and Center for Integrated Animal Genomics, Iowa State University, 2255 Kildee Hall, Ames, IA, 50011, USA

**Keywords:** Human, Genome, microRNA, Duplication, Evolution

## Abstract

**Background:**

Most studies on the origin and evolution of microRNA in the human genome have been focused on its relationship with repetitive elements and segmental duplications. However, duplication events at a smaller scale (<1 kb) could also contribute to microRNA expansion, as demonstrated in this study.

**Results:**

Using comparative genome analysis and bioinformatics methods, we found nine novel expanded microRNA families enriched in short duplicated sequences in the human genome. Furthermore, novel genomic regions were found to contain microRNA paralogs for microRNA families previously analyzed to be related to segmental duplications. We found that for microRNA families expanded in the human genome, 14 families are specific to the primate lineage, and nine are non-specific, respectively. Two microRNA families (hsa-mir-1233 and hsa-mir-622) appear to be further expanded in the human genome, and were confirmed by fluorescence *in situ* hybridization. These novel microRNA families expanded in the human genome were mostly embedded in or close to proteins with conserved functions. Furthermore, besides the Alu element, L1 elements could also contribute to the origination of microRNA paralog families.

**Conclusions:**

Together, we found that small duplication events could also contribute to microRNA expansion, which could provide us novel insights on the evolution of human genome structure and function.

## Background

Although only ~22 nucleotides long, microRNA are vital to the developmental process of animals and plants through post-transcriptional gene regulation. The efficiency of microRNA transcription and stability is regulated in a tissue-specific manner [1-4]. After processing from precursor microRNA molecules, single-stranded microRNA can bind to 3'-UTR of messenger RNA via its seed region, in turn affecting mRNA translation or stability [1,5]. When aberrantly expressed, microRNA can lead to the progression of certain diseases [6,7]. They also participate in pathogen-host interaction [8], and exert systemic effects through intercellular trafficking [9]. Due to its significant roles in a wide variety of biological processes, genome evolution of microRNA structure and function have been studied extensively [1,2,10-18].

Both duplicated sequence fragments and repetitive elements in the genome could contribute to the expansion of microRNA families [10-18]. Species-specific microRNA expansion could have functional importance, and it has been demonstrated recently that an expanded microRNA cluster is fundamental to the maintenance of embryonic stem cell development in mice [13]. However, most duplication events studied for microRNA evolution are segmental or tandem duplications [15-17]. Segmental duplications are tremendously important in elucidating the evolution of protein-coding genes, and their roles in providing novel genomic mechanisms to cope with selection pressure and environmental changes [19-24]. However, segmental duplications are defined as > 1 kb and > 90% identity, and computational methods are designed accordingly and limited in studying only these sequences. Thus, small-scale duplication events are not covered, but which could also play important roles in the evolution of small molecules, especially for microRNA. The relevance and importance of these small duplication events and their relationship with microRNA evolution has not been reported.

Here, we used a systematic approach, and found that small duplication events in the human genome could also contribute to microRNA expansion. In total, nine novel microRNA families were found to be expanded in the human genome, and additional new genomic regions were discovered to be related to the expansion of microRNA families reported previously. We found that novel microRNA family expanded in the human genome are located close to proteins with conserved function, and confirmed two of the microRNA expansion events by fluorescence *in situ* hybridization. These results could render us novel insights on the evolution of human genome structure and function.

## Results

### Novel expanded microRNA families in the human genome

To explore the relationship between short duplicated genomic sequences and the expansion of certain microRNA families in the human genome, we used repeat-masked reference genome for pairwise comparison (see Methods), since microRNA associated with repetitive elements have been well characterized [16-18]. We focused on detecting microRNA paralogs enriched in short duplication events (<1 kb) in the human genome, which were overlooked previously by studies on the role of segmental duplications (emerged recently, >1 kb and > 90% identity) to the origin and evolution of microRNA families [10-17].

In total, nine novel microRNA families were found to be enriched in short duplicated fragments (Additional file 1). We detected 26 microRNA families, previously found to be related to segmental duplications [16], in which new microRNA paralogs could also be enriched in short genomic fragments (Additional file 2).

**Figure 1:**
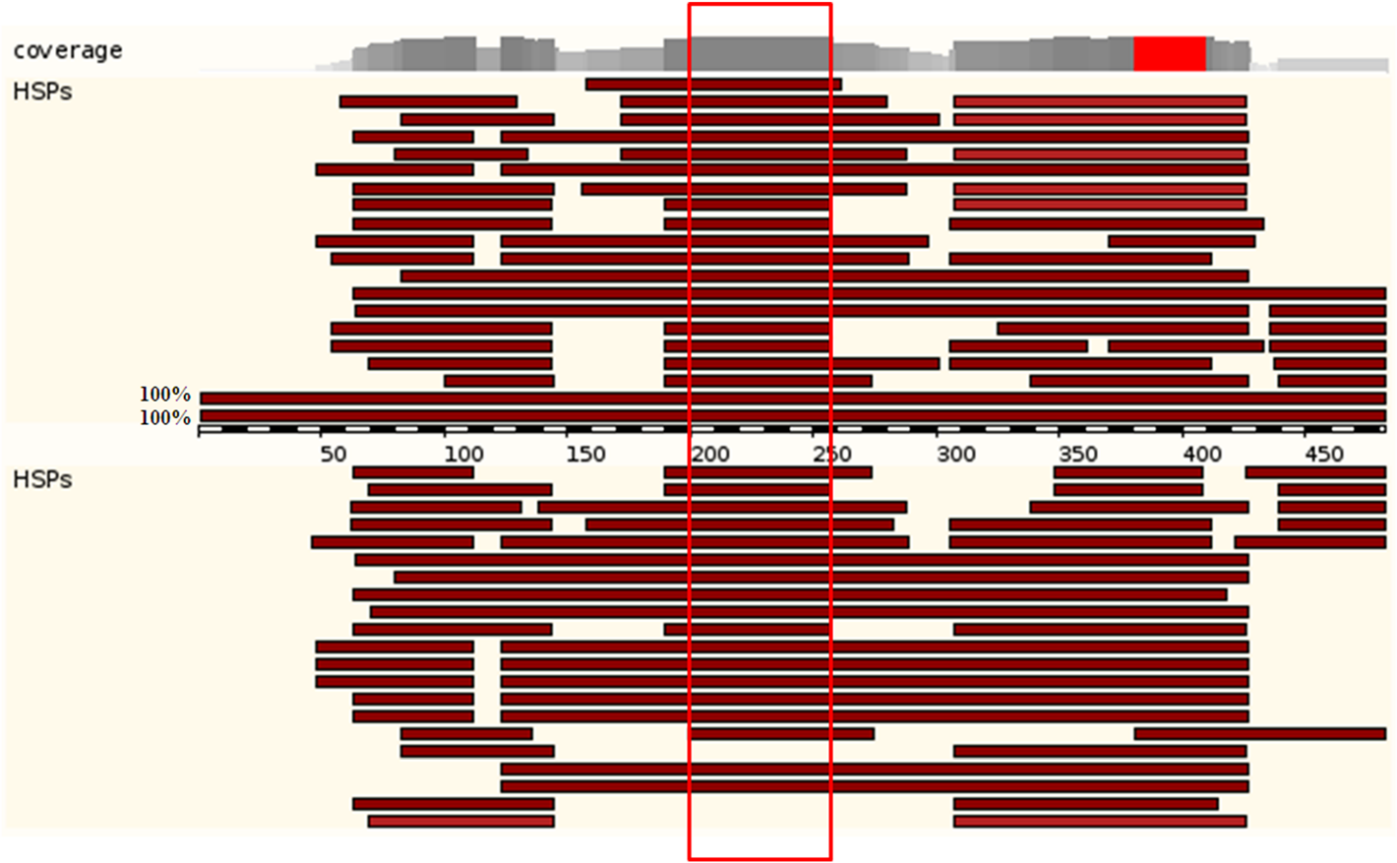
**Detailed local genomic sequence analyses reveal new patterns of expanded microRNA families (hsa-mir-1233).** Typical Ensembl sequence alignment picture reveals that novel paralogs will not be found if using only segmental duplication analysis, since it will detect only > 1 kb fragments of > 90% identity. In the region around 200–258 bp corresponding to conserved sequences to two microRNAs (in red frame), hsa-mir1233-1 and hsa-mir1233-2 (100% identity as indicated), we can see multiple genomic fragments, which are short and isolated, but highly conserved (percentage of identity can be found in Additional file 2). These genomic regions could contain potential microRNA paralogs. HSPs *=* high scoring pairs.

For the nine novel microRNA families, the average identity score is 89.1%, slightly lower compared to microRNA enriched in segmental duplications (90.4%), and far lower than those already deposited in miRBase (99%). These nine novel microRNA families are derived from small duplications, with an average length around 430 bp, which could be the reason they were overlooked by previous segmental duplication analysis. Furthermore, through detailed analyses on local genomic regions, microRNA families previously identified by segmental duplication analyses to be expanded in the human genome could have new paralogs (Additional file 2, Figure 1). For instance, for the microRNA family hsa-mir-1233, multiple isolated and conserved genomic fragments shorter than 1 kb could contain potential microRNA paralogs (Figure 1).

Furthermore, our methods were able to detect 4 mitochondria-related microRNA families (Additional file 3), and 20 other microRNA families already deposited in miRBase (Additional file 4). However, three of the four mitochondria-related microRNA families, hsa-mir-1974, hsa-mir-1977, and hsa-mir-1978, were not further curated in miRBase, since they overlap with transfer-RNA (t-RNA) sequences. Despite the exclusion of these three microRNA in the miRBase, experimental evidence suggests that microRNA can be derived from t-RNA, and still demonstrate biological functions [25,26].

**Figure 2:**
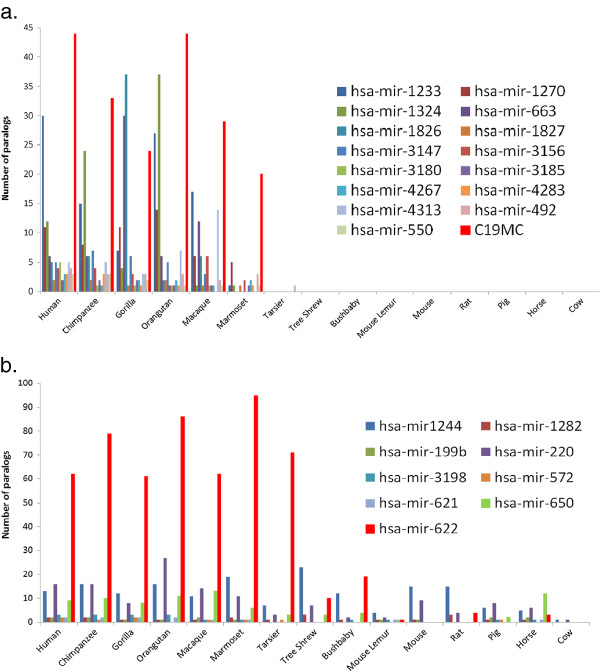
**Primate lineage-specifically expanded microRNA families. a**. 16 primate-specific families. The microRNA family on human chromosome 19 (C19MC) is the largest cluster identified previously. The hsa-mir-1233 mainly clustered on chromosome 15 expanded further in the human genome. **b**. Primate-nonspecific families (9). The hsa-mir-622 family seems to be further expanded in the primate lineage.

To see if these microRNA families expanded in the human genome were specific to the primate lineage, we did further comparison to 14 other mammalian genomes (see Methods). We found 16 primate-specific microRNA families (Figure 2a), and the microRNA cluster on chromosome 19 (C19MC) is the largest primate-specific cluster and may contribute to human reproduction, which has already been examined in detail [10,11,27]. The hsa-mir-1233 family appears to be further expanded in the human genome, compared to other primates. Furthermore, four novel microRNA families detected here were also specific to primate genomes (hsa-mir-1826, hsa-mir-1827, hsa-mir-3185 and hsa-mir-492) (Figure 2a). In addition, nine microRNA families were found to be nonspecific to primate genomes, in which four of them were novel (hsa-mir-622, hsa-mir-220, hsa-mir-199b and hsa-mir-1282) (Figure 2b). Investigation into the hsa-mir-622 family reveals that it further expanded in primates (Figure 2b), but with lower sequence conservation rates (88.0%).

### Patterns of emergence of microRNA paralogs

We examined the location of these microRNA paralogs relative to protein-coding genes, the sequence conservation rate, and the flanking genomic sequences as well, to see if there exist certain patterns related to the expansion of these detected microRNA paralog families.

**Figure 3:**
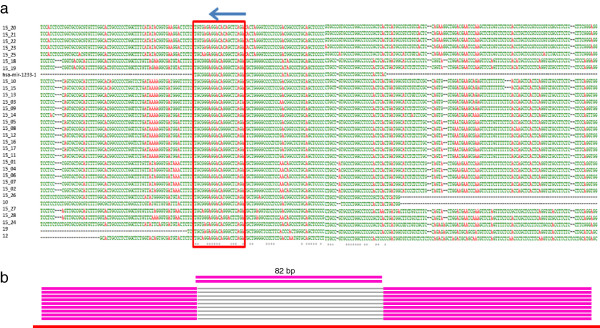
**Patterns of microRNA paralog emergence. a**. Multiple sequence alignment for microRNA paralog family hsa-mir-1233. Red frame and arrow indicate the mature microRNA sequences and their orientations. The seed regions (2–8) are not highly conserved. **b**. cDNA evidence. Typical blast results show the alignment of duplicate (red lines) with expressed transcripts (pink lines) (Additional file 6). The 82 bp fragment containing the mature microRNA sequence cannot align to mRNA or non-coding RNA sequences in the public database, which could be spliced out.

Nearly all of the duplicated genomic fragments containing microRNA paralogs were localized in intronic regions of protein-coding genes or non-coding transcripts, and only two of them were found in the 3^′^-untranslated region (UTR), which is in agreement with the origin of novel microRNA proposed previously [28] (Additional file 5). Evidence of cDNA expression for microRNA paralogs was found through public database searching (NCBI, see Methods). Furthermore, novel microRNA families expanded in the human genome were found to be embedded in proteins (host genes) mostly with conservative functions, such as tubulins and keratins (Additional file 5). For instance, the hsa-mir-1233 family was found to be related to golgi autoantigen, golgin subfamily a, 6 pseudogenes, which are expressed in fetal brain (hypothalamus) and embryonic stem cells (Additional files 5 and 6). Interestingly, we found that for the hsa-mir-1233 family, the conserved mature microRNA sequences (an 82-bp fragment) in all the paralogs were found to be missing from the cDNA sequences deposited in the database (Figure 3b). These 82-bp fragments in the paralogs could potentially be spliced out after processing precursor mRNAs, and subsequently function as microRNA.

Direct analyses on microRNA paralog sequence features in all expanded microRNA families found that mature microRNA sequences and the seed regions of microRNA paralogs could be under the influence of different selective forces [29]. For instance, in two microRNA families, hsa-mir-1233 and hsa-mir-1244 (Figure 3, Additional file 7), we can clearly see that most of the microRNA paralogs had nearly identical mature sequences, while others demonstrate dissimilar features. These sequence changes could affect the stability of the stem-loop structure of the microRNA paralogs. Further examination on the flanking genomic regions of these microRNA families found they are highly conserved (Figure 3, Additional file 7).

Furthermore, the analysis on repetitive elements in the flanking genomic regions may contribute to the understanding of the origin of these microRNA families. Repetitive elements, such as Alu elements, were discovered previously to potentially affect the expansion of certain microRNA clusters as well as the genes located in duplicated genomic segments [11,30]. We found that Alu elements are also enriched in the flanking regions of microRNA paralogs (ANOVA) (Additional file 8). Surprisingly, the L1 elements have also been enriched in these sequences, which implied that they could also function in the evolutionary path of formation of certain microRNA paralogs. Furthermore, L1 elements had the highest percentage (21%), but not significantly different with AluS elements (*t*-test, P > 0.05).

### Validation of expansion of two microRNA families

**Figure 4:**
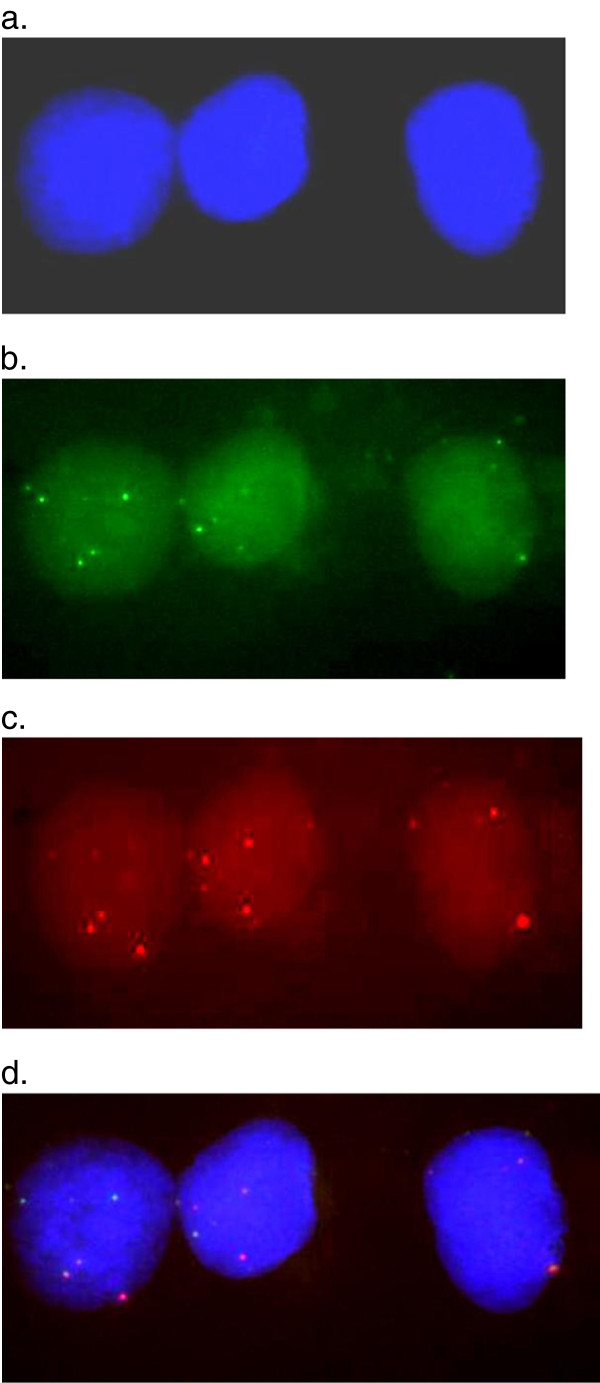
**Fluorescence *in situ *hybridization (FISH) performed using duplex-probes (×1000). a**. Hoechst33342 (Blue). **b**. hsa-mir-622 family (Green). **c**. hsa-mir-1233 family (Red). **d**. The merged signals indicate two probes hybridize to genomic sequences close to each other, which is in agreement with the computational prediction results.

We further confirmed our results for two microRNA families found to be expanded in the human genome, using fluorescence *in situ* hybridization (FISH) (Figure 4). One of them was identified previously (hsa-mir-1233), and one novel microRNA family (hsa-mir-622) detected in this study. We can see that both probes hybridize to multiple locations in the human genome (Figure 4). Furthermore, our computational methods detected that both miRNA families have paralogs on the same chromosomes, 8, 9, 12 and Y (Additional files 1 and 2), which is evident in the merged hybridization signals being nearby each other (Figure 4).

## Discussion

Using information provided by short duplication events in the human genome, we discovered novel microRNA paralogs for different microRNA families, which could have fundamental importance for human biology and the study of complex phenotypic traits. However, since the number of microRNAs deposited in the public database are far less than predicted, new microRNA paralogs and clusters may still exist in the human genome. Furthermore, this method can be extended for other non-coding RNAs, which can potentially reveal interesting patterns of specific important duplications in the human genome.

The study of microRNA from a comparative genomics standpoint is not without some limitations. The majority of the computational prediction methods use either precursor or mature microRNA sequences, combined with the examination of predicted secondary-structure [31]. The drawback of this approach is that only highly conserved potential microRNAs within or across species can be discovered. Experimental methods to date have only sampled limited tissues at few time-points, which will restrict the identification of novel microRNA [32]. Even though high-throughput small RNA sequencing has led to the identification of numerous novel microRNAs, the number and speed of discovery are still limited [33]. Our methods could to some degree help the identification and interpretation of novel microRNA paralogs in the human genome.

Alu elements have been proposed to be involved in the propagation of microRNA clusters and segmental duplications [11,30]. We also found in this study that AluS is the most abundant subfamily (17%), followed by AluJ (8%) and AluY (4%) (Additional file 8), which indicates that microRNA expansion events discovered here could follow a similar evolutionary path as reported in [11]. Furthermore, we provide evidence here that in addition to Alu elements, L1 repetitive elements are also associated with duplication of specific microRNA sequences. While numerous molecular mechanisms could be hypothesized for microRNA expansion, there remains a strong association that specific repetitive elements may have played important roles in the evolution of microRNA containing loci in humans. These duplicated paralogs could obtain partial and/or novel functions or exert dosage effects, acquire novel regulatory elements for tissue-specific expression, possess modifications in seed sequence resulting in novel microRNAs, and potentially beneficial or detrimental to certain individuals or a specific population [29]. The conserved flanking sequences indicate that similar regulatory mechanisms could be involved in the tuning of these paralogs, but the changed sequences of the mature microRNA paralog could target different sets of genes and affect different gene clusters. However, detailed functional analysis is still needed to understand the outcome of the duplicated microRNA molecules [13].

We found that novel microRNA could potentially be generated through the processing of pseudogene transcripts. These expanded microRNA families in the human genome could provide novel insights on the evolution and regulation of the human genome and its interaction with the environment. Particularly, these expanded microRNA families could add another level of complexity into the regulatory network formed among messenger RNAs, transcribed pseudogenes, long noncoding RNAs, through the amplification of microRNA abundance [34].

Limited amounts of population resequencing data restricted our understanding of intra-species variation regarding one of the structural variations, the expansion of microRNA family, which has important practical implications (e.g. disease genetics). However, the rapid developments in sequencing technology will alleviate this problem in the near future, as already shown in the 1000 genomes project [35-37]. Further detailed analysis on the association of microRNA expansion with features unique to each ethnic group will potentially reveal their biological importance in human diversity.

## Conclusions

Taken together, we found that small duplication events in the human genome may contribute to microRNA expansion, which could provide novel insights on the evolution of genome architecture, in addition to the development of human diseases.

## Methods

### Sequence alignments

The whole procedure for the analysis can be found in Additional file 9. The human genome and 25 other animal genome sequences with repetitive elements masked were downloaded from Ensembl (Additional file 10). We searched for genomic duplications by Megablast using default parameters [38]. All duplicated fragments were kept without restriction on length and identity of the sequence alignments, which is different from the detection methods for segmental duplications (length >1 kb and identity >90%) [30].

### Enrichment of duplicated fragments containing microRNAs

Coordinates of human microRNAs were retrieved from miRBase (GRCh37) [39], and used to enrich for those duplicated genomic fragments overlapping with microRNAs using in-house Perl scripts. The extracted final coordinates were compared again to those of microRNAs deposited in miRBase, to find potential novel duplicated microRNAs, as well as their genomic locations with regard to exonic, intronic or untranslated regions of known genes.

### Paralogs and orthologs of duplicated microRNAs

To search for paralogs and orthologs of microRNAs duplicated in the human genome, we retrieved the regional genomic sequences with repetitive sequences masked surrounding a representative microRNA deposited in miRBase, 3,000 base pairs (kb) upstream and downstream. The sequences were used to compare to the human genome sequence using Megablast, and an iterative procedure was used to enrich for genomic sequences related to microRNA paralogs. Furthermore, the retrieved genomic sequences were used to detect orthologs in other species using Megablast, including nine primates (*Pan troglodytes, Pongo pygmaeus*, *Gorilla gorilla, Macaca mulatta*, *Callithrix jacchus*, *Tarsius syrichta*, *Tupaia belangeri*, *Microcebus murinus*, and *Otolemur garnettii*), two rodents (*Rattus norvegicus* and *Mus musculus*), and three domestic livestock species (*Equus caballus*, *Bos taurus* and *Sus scrofa*) (Additional file 10). cDNA evidence was also searched in NCBI by using the retrieved genomic sequences containing microRNAs detected to be expanded in the human genome.

### Repetitive elements in the flanking genomic regions and microRNAs

To detect human microRNAs in miRBase overlapping with the repetitive elements, we retrieved the genomic positions of the repetitive elements in the human genome from the UCSC genome table browser (group: variation and repeats, track: RepeatMasker) [40], and compared results to the coordinates of human microRNAs in miRBase. To further explore the relationship of repetitive elements with the origin of duplicated microRNA paralogs, the coordinates of repeats distributed in the regions of microRNA paralogs (upstream and downstream 5 kb regions) were compared and examined. Fractions of repetitive elements to the selected genomic sequences were calculated by their nucleotide lengths, and enrichment test was performed using Chi-square test, by comparing to the fraction of repetitive elements in the whole human genome. Secondary structure of the microRNA precursor was predicted using RNAfold [41].

### Fluorescence *in situ* hybridization

To validate the results obtained by computational prediction, we selected two probes for the two microRNA families (hsa-mir-1233 and hsa-mir-622), and labeled them with TAMRA (red) and FITC (green), respectively (Additional file 11). FISH was performed on a normal primary neonatal dermal fibroblast cell line of European origin (PCS-201-010 from ATCC® Primary Cell Solutions™) following standard procedures (Creative™ Biolabs) [42]. Due to the short probe length (~1 kb), we optimized the hybridization condition several times, until consistent results were obtained.

## Competing interests

The authors declare no competing interests.

## Authors’ contributions

ZQD, MFR and JWR designed the research. ZQD and CXY performed the experiments. ZQD and CXY analyzed data. ZQD, MFR and JWR wrote the manuscript. All the authors read and approved the final manuscript.

## Supplementary Material

Additional file 1 Nine novel microRNA families expanded in the human genome. Click here for file

Additional file 2 Six microRNA families previously found in the human genome. Click here for file

Additional file 3 Four expanded microRNA families related to mitochondria. Click here for file

Additional file 4 Twenty expanded microRNA families deposited in the human genome. Click here for file

Additional file 5 Expanded microRNA families and protein-coding genes. Click here for file

Additional file 6 cDNA evidence for miRNA paralogs (hsa-mir-1233 family). Click here for file

Additional file 7 Potential novel mechanism of emergence for microRNA family hsa-mir-1244. Click here for file

Additional file 8 Repeat elements surrounding duplicated miRNA paralogs. Click here for file

Additional file 9 Flowchart for computational analysis on animal microRNA expansion. Click here for file

Additional file 10 25 Genome sequences screened for miRNA expansion. Click here for file

Additional file 11 Two probes used for FISH analysis. Click here for file
