# Supplementary material for: Novel microRNA families expanded in the human genome
Source: BMC Genomics. 2013 Feb 12;14:98. doi: 10.1186/1471-2164-14-98 (PMC3602292; doi:10.1186/1471-2164-14-98)

Additional file 7. Potential novel mechanism of emergence for microRNA family hsa-mir-1244

1. Multiple sequence alignment for microRNA family hsa-mir-1244. Similarly, red frame and arrow indicate the mature microRNA sequences and their orientations. The seed regions (2-8) are highly conserved, except for a paralog on chromosome X (seed region deleted) (the small red frame).
2. Secondary structure of the microRNA precursor (hsa-mir-1244-1) in miRBase on chromosome 2. Its free energy of the thermodynamic ensemble is -18.27 kcal/mol. Arrow indicates sequences deleted in the paralogous sequence on X chromosome, which could disrupt the hair-pin structure of pre-microRNA.
3. Unstable secondary structure, predicted when using directly the paralogous sequences on X chromosome. The free energy of the thermodynamic ensemble is only -12.13 kcal/mol.
4. However, when we extended on both sides of the flanking sequences, and selected them to predict the secondary structure, the free energy of the thermodynamic ensemble decreases to -20.20 kcal/mol, and more stable.

a.

2 ATCTTATTCCGAGCATTCCAGTAACTTTTTT-GTGTATG--TACTTAGCTGTACTATAAGTAGTTGGTTTGTATGAGATGGTTAAAAA

X ATCTTATTCCGAGTGTTCCAGTGTCTTTTTT-GTATATG--AACTTAGCGGTACTATAAGT-------TTGTATGAGATGGTTAAAAA

13 ATCTTATTCTGAGCATTCCAGTAACTTTTTT-GTGTATG--TACTTAGCTGTACTATAAGGAGTTGGTTTGTATGAGATGGTTAAAAA

6 ----------GAGCATTCCAGTAGCTTCTTTAGTGTATG--TAGTTAGTTGTACCATAAGTAGTTGGTTTGTGTGAGATGGTTAAAAA

7 ---------CGAGCATTCCAGTAACTTTTTTGG-GTATG--TACTTAGTTGTACCATAAGTAGTTGGTTTGTGTGAGATGGTTAAAAA

12 ATCTTATTCCGAGCATTCCAGTAACTTTTTT-GTGTATG--TACTTAGCTGTACCACAAGTAGTTGGTTTGTATGAGATGGTTAAAAA

5 ATCTTATTCCGAGCATTCCAGTAACTTTTTT-GTGTATG--TACTTAGCTGTACTATAAGTAGTTGGTTTGTATGAGATGGTTAAAAA

14 ATCTTATTCCGAGCATTCCAATAACTTTTTT-GTGTATG--TACTTAGCTGTACTATAAGTAGTTGGTTTGTATGAGATGGTTAAAAA

hsa-mir-1244-1 ATCTTATTCCGAGCATTCCAGTAACTTTTTT-GTGTATG--TACTTAGCTGTACTATAAGTAGTTGGTTTGTATGAGATGGTTAAAAA

9-01 ATCTTATTCCCACCATTGCAGTAACTTTTTT-GTGTATG--TACTTAGCTGTACTATAAGTAGTTGGTTTATATGATCTAGTTAAAAA

9-02 ATCTTATTCCCACCATTGCAGTAACTTTTTT-GTGTATG--TACTTAGCTGTACTATAAGTAGTTGGTTTATATGATTTGGTTAAAAA

20 ATCTTATTCTGAGCATTCCAGTAACTTTTTT-GTGTGTGCGTACTTAGCTGTACTATAAGTAGTTGGTTTGTATGAGATGGTTAAAAG

3 ATCTTATTCTGAGCATTCCAGTAACTTTTTT-GTGTATG--TACTTAGCTGTACTACAAGTAGTTGGTTTGTATGAGATGGTTAAAAA

* ** ** * *** *** * * ** * **** **** * *** ** * *** * *******

b. c. d.


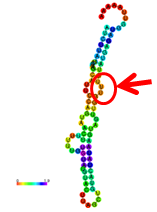

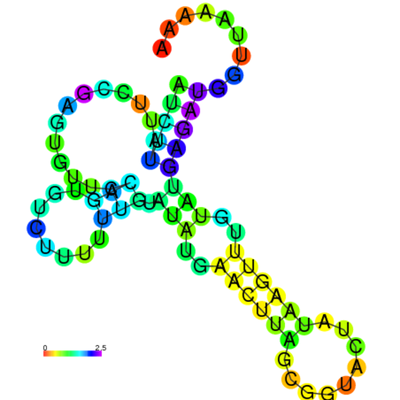

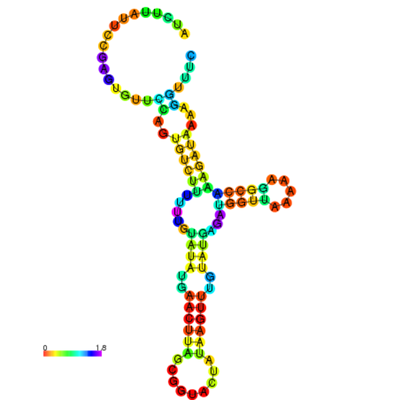

Supplement: Additional file 7 — Potential novel mechanism of emergence for microRNA family hsa-mir-1244. [file 1471-2164-14-98-S7.docx]
