# Supplementary material for: Novel microRNA families expanded in the human genome
Source: BMC Genomics. 2013 Feb 12;14:98. doi: 10.1186/1471-2164-14-98 (PMC3602292; doi:10.1186/1471-2164-14-98)
Supplement: Additional file 8 — Repeat elements surrounding duplicated miRNA paralogs. [file 1471-2164-14-98-S8.docx]

Additional file 8: Repetitive elements surrounding duplicated miRNA paralogs

| Family | SINE | | | | LTR | hAT | TcMar | LINE | |
| --- | --- | --- | --- | --- | --- | --- | --- | --- | --- |
|  |  | Alu |  | MIR |  |  |  | L1 | L2 |
|  | AluJ | AluS | AluY |  |  |  |  |  |  |
| hsa-mir-1233 | 48 | 77 | 25 | 91 | 11 | 39 | 14 | 39 | 27 |
| hsa-mir1244 | 33 | 64 | 12 | 12 | 37 | 10 | 11 | 42 | 27 |
| hsa-mir-1270 | 7 | 61 | 14 | 0 | 11 | 1 | 0 | 65 | 1 |
| hsa-mir-1282 | 1 | 1 | 0 | 3 | 3 | 4 | 0 | 7 | 2 |
| hsa-mir-1324 | 8 | 14 | 1 | 4 | 2 | 0 | 0 | 22 | 2 |
| hsa-mir-1826 | 3 | 1 | 2 | 0 | 0 | 0 | 0 | 0 | 0 |
| hsa-mir-1827 | 2 | 4 | 0 | 0 | 3 | 2 | 0 | 5 | 3 |
| hsa-mir-650 | 5 | 11 | 4 | 13 | 5 | 6 | 1 | 22 | 10 |
| C19MC | 11 | 30 | 8 | 20 | 29 | 0 | 5 | 47 | 7 |
| hsa-mir-663 | 4 | 2 | 1 | 7 | 3 | 2 | 2 | 3 | 1 |
| hsa-mir-199b | 3 | 4 | 0 | 4 | 2 | 0 | 0 | 3 | 2 |
| hsa-mir-220 | 17 | 39 | 9 | 17 | 46 | 8 | 2 | 38 | 7 |
| hsa-mir-3147 | 5 | 12 | 1 | 3 | 3 | 1 | 0 | 26 | 0 |
| hsa-mir-3156 | 0 | 5 | 2 | 1 | 2 | 2 | 2 | 0 | 2 |
| hsa-mir-3180 | 10 | 23 | 3 | 6 | 1 | 2 | 0 | 14 | 1 |
| hsa-mir-3185 | 0 | 1 | 0 | 0 | 4 | 0 | 0 | 12 | 0 |
| hsa-mir-3198 | 3 | 5 | 1 | 2 | 2 | 1 | 0 | 9 | 3 |
| hsa-mir-4267 | 1 | 3 | 2 | 1 | 10 | 0 | 0 | 9 | 0 |
| hsa-mir-4283 | 4 | 5 | 1 | 3 | 2 | 3 | 0 | 0 | 3 |
| hsa-mir-4313 | 10 | 16 | 0 | 5 | 7 | 2 | 3 | 16 | 11 |
| hsa-mir-492 | 3 | 7 | 1 | 9 | 3 | 1 | 1 | 30 | 1 |
| hsa-mir-550 | 1 | 3 | 0 | 2 | 0 | 0 | 0 | 0 | 0 |
| hsa-mir-572 | 1 | 3 | 2 | 0 | 4 | 0 | 0 | 0 | 0 |
| hsa-mir-621 | 1 | 2 | 0 | 0 | 0 | 0 | 0 | 1 | 1 |
| hsa-mir-622 | 86 | 182 | 43 | 79 | 178 | 48 | 31 | 297 | 78 |
| No. | 267 | 575 | 132 | 282 | 368 | 132 | 72 | 707 | 189 |
| Fraction | 0.08 | 0.17 | 0.04 | 0.08 | 0.11 | 0.04 | 0.02 | 0.21 | 0.06 |
| Fraction in the genome | 0.03 | 0.07 | 0.01 | 0.04 | 0.18 | 0.04 | 0.03 | 0.16 | 0.03 |
