# Supplementary material for: Novel microRNA families expanded in the human genome
Source: BMC Genomics. 2013 Feb 12;14:98. doi: 10.1186/1471-2164-14-98 (PMC3602292; doi:10.1186/1471-2164-14-98)
Supplement: Additional file 9 — Flowchart for computational analysis on animal microRNA expansion. [file 1471-2164-14-98-S9.docx]

Human genome sequences

Coordinates of human miRNAs in miRBase

Coordinates of duplicates

Enrichment of duplicated miRNAs with overlapped coordinates

Extract genomic sequences plus flanking sequences (3kb on both sides)

To find human miRNA orthologs

25 other animal genome sequences

megablast

megablast

megablast

Phylogenetic analysis, repetitive elements, cDNA evidence, functional analysis, etc.

To find human miRNA paralogs

**Additional file 9: Flowchart of computational analysis on animal microRNA expansion**
