# Supplementary material for: Novel microRNA families expanded in the human genome
Source: BMC Genomics. 2013 Feb 12;14:98. doi: 10.1186/1471-2164-14-98 (PMC3602292; doi:10.1186/1471-2164-14-98)
Supplement: Additional file 10 — 25 Genome sequences screened for miRNA expansion. [file 1471-2164-14-98-S10.docx]

Additional file 10: 25 Genome sequences screened for miRNA expansion

| Species name | Common name | Order |
| --- | --- | --- |
| *Class Aves* |  |  |
| *Gallus gallus* | Chicken | Galliformes |
| *Taeniopygia guttata* | Zebrafinch | Passeriformes |
|  |  |  |
| *Class Mammalia* |  |  |
| *Microcebus murinus* | Grey mouse lemur | Primates |
| *Tarsius syrichta* | Tarsier | Primates |
| *Otolemur garnettii* | Bush baby | Primates |
| *Callithrix jacchus* | Common marmoset | Primates |
| *Macaca mulatta* | Rhesus macaque | Primates |
| *Papio hamadryas* | Hamadryas baboon | Primates |
| *Pongo pygmaeus* | Orang-utan | Primates |
| *Gorilla gorilla* | Gorilla | Primates |
| *Pan troglodytes* | Chimpanzee | Primates |
| *Homo sapiens* | Human | Primates |
|  |  |  |
| *Bos taurus* | Cow | Artiodactyla |
| *Sus scrofa* | Pig | Artiodactyla |
|  |  |  |
| *Equus caballus* | Horse | Perissodactyla |
|  |  |  |
| *Canis familiaris* | Domestic dog | Carnivora |
|  |  |  |
| *Mus musculus* | Mouse | Rodents |
| *Rattus norvegicus* | Brown rat | Rodents |
|  |  |  |
| *Oryctolagus cuniculus* | European rabbit | Lagomorpha |
| *Loxodonta africana* | African elephant | Proboscidea |
|  |  |  |
| *Monodelphis domestica* | Oppossum | Didelphimorphia |
|  |  |  |
| *Ornithorhynchus anatinus* | Duck-billed platypus | Monotremata |
|  |  |  |
| Class Actinopterygii |  |  |
| *Danio rerio* | Zebrafish | [Scorpaeniformes](http://www.catalogueoflife.org/browse/tree/id/2337862) |
| *Gasterosteus aculeatus* | Stickleback | [Gasterosteiformes](http://www.catalogueoflife.org/browse/tree/id/2340536) |
| *Tetraodon nigroviridis* | Tetraodon | [Tetraodontiformes](http://www.catalogueoflife.org/browse/tree/id/2344480) |
| *Oryzias latipes* | Medaka | [Beloniformes](http://www.catalogueoflife.org/browse/tree/id/2341885) |
