# Supplementary material for: Novel microRNA families expanded in the human genome
Source: BMC Genomics. 2013 Feb 12;14:98. doi: 10.1186/1471-2164-14-98 (PMC3602292; doi:10.1186/1471-2164-14-98)
Supplement: Additional file 11 — Two probes used for FISH analysis. [file 1471-2164-14-98-S11.docx]

**Additional file 11: Two probes used for FISH**

1. hsa-mir-1233 (TAMRA labeled)

AGGGATGTTGCACACCCTACCTTCCTCTCCTCCTTGTCCTGGGCCAGCCTGATGATGGCCTCCTCCCGGTGACGCATCTTTGGCACTGTCCCCTGGCTCTGTTAGAAGGCGATGTACTTTCCTGCGGGAGGACAGGGCTCAGACGCTGGGGCCCCTCCCATAGCCCTGCAGCTCCCCCTGCCGTGCCCTGGCCTCCCACTCACTGATGGCATCTCTCTCTCCGGTACTGGATGAATCCAAGTTCCAGTTTCTCCACATGCTCCCTCAGGTCCGCCTTCTCCTCCAGGAGGTCCATAAGGCCGCTCTGGAGCCAAAATAATGGGGTCACATCTCAGCAGCGACCTGCCCCAGCCCTGCCCTTCTTGGCCCATGCTAGGACTCACTCCCCTCCAGCTTCTCCATGACTTCCTGCAGGGCCCGGAGGGTCTCCCCACTCACAGACTCGCCCCCAGTCCCTGAGGCTGGGACTGCTGCCTCTGGCTCCTTCTCGGCCGAGGTGAGCGTCTCCATCACCTGGCCCAGCTTCTCCTGCAGCTCCTTTACTTGCTGCTCCAACTGCAGTGCGCTCTTGTTCTCGTGCTTCTGGACAGAGAGAAGCAATCAGCAGCCACCCACTGCAGCTGGAGACCCCAGAACTTGGTGTCTGCCTCCCATGGCACCGGGAAGGGTGGAGCCAAGTTAGAAAAATACTCTCCTCTCTCCCACAGCCACCAGAGCAAAGCTCTGGCTCACAGGTGCCTTTGGAAGTAATATTTCATGTGAGGGCTAC

1. hsa-mir-622 (FITC labeled)

GGGAGCACCTGGAGAAGACAGGACCCCAGGTCAGAGACTGGGTCCATTATTTCAAGACCATCGAGGACCTGAGGGCTCATATCTTCACAAATACTGTGGACAATGTCCACATTGTTCTGCAGATCGACAATGCCCATCTTGCTGCTGATGCCTTTAGAGTCAAGTATGACACAGAGCTGGCCATGCGCCAGTCTGTGGAGAGCGACATCTATGGGCTCTGCAAGGTCATTGATGACACCAGTGTCACTCAGCTGCAGCTGAAGACAGAGATCGAGACTCTCAAGGAGGAGCTGCTCTTAATGAAGAAGAACCATGAAGAGGAAGTAAAAGGCCTACAAGCCCAGATTGTCAGCTCTGGTTTGATCTTGGAGGTAGATGCCCCCAAATCTCAGGACCTTGCCAAGATCATGGCAGACATCTGGGCCCAATATGACGAGCTGGCTCGGAAGAACTGAGAAGGGCTGGACAAATACTGGTCTCAGCAGATTAAGGAGAGTACCACAGTGGTCACCATGCAGTTGGCCAAGGTTGGAGCTGTTGAGATGATGCTCTCAACAGATGAGCTGTAACATACAGCCCAGTCCTCGGAGACTGACCTGGACTCCATGAGAAATCTGAAGGCCAGCTTGGAGAACAGCCTGAGGGAGGTGGAGGCCTGGTACACCCTGCAGATGGAGCAACTCAACGGGATCCTGCTGCACCTGGAGTTGGAGTTGACACAGACCCGGGCAGAGGGGCAGCACCAGGCCCAGGAGTAGGAGGCCCTGCTGAACATTAAGGTCAAGCTGGAGGCTGAGATTGCCATGTATCACTGCCTGCCAGAAGACATCAAGGACTTCAGTCTTGGTGATGCCTTGGACAGCAGTAACTCCATGCAAACCATCCAAAAGACCACCACCCGCCAGACAGTGGATGGCAAAGTGGTGTCTGAGATCAATGACACCAAAGTTCTGAGACATTAAGCCAGCAGAAGCAGGGTACTCTCTGGGGAGCTGGAAGCCAATAAAAAGTTCAGAGG
